# Risk of dementia in patients with toxoplasmosis: a nationwide, population-based cohort study in Taiwan

**DOI:** 10.1186/s13071-021-04928-7

**Published:** 2021-08-28

**Authors:** Hung-Yi Yang, Wu-Chien Chien, Chi-Hsiang Chung, Ruei-Yu Su, Chung-Yu Lai, Chuan-Chi Yang, Nian-Sheng Tzeng

**Affiliations:** 1 Division of Clinical Pathology, Department of Pathology, Tri-Service General Hospital, School of Medicine, National Defense Medical Center, Taipei, Taiwan; 2 Department of Medical Research, Tri-Service General Hospital, National Defense Medical Center, Taipei, Taiwan; 3 School of Public Health, National Defense Medical Center, Taipei, Taiwan; 4 Graduate Institute of Life Sciences, National Defense Medical Center, Taipei, Taiwan; 5 Taiwanese Injury Prevention and Safety Promotion Association, Taipei, Taiwan; 6 Graduate Institute of Aerospace and Undersea Medicine, School of Medicine, National Defense Medical Center, Taipei, Taiwan; 7 Department of Psychiatry, Taoyuan Armed Forces General Hospital, Taoyuan, Taiwan; 8 Department of Psychiatry, Tri-Service General Hospital, School of Medicine, National Defense Medical Center, Taipei, Taiwan; 9 Student Counseling Center, National Defense Medical Center, Taipei, Taiwan

**Keywords:** *Toxoplasma gondii*, Toxoplasmosis, Dementia, Taiwan

## Abstract

**Background:**

Approximately 25–30% of individuals worldwide are infected with *Toxoplasma gondii* (*T. gondii*), which is difficult to detect in its latent state. We aimed to evaluate the association between toxoplasmosis, the risk of dementia, and the effects of antibiotics in Taiwan.

**Methods:**

This nationwide, population-based, retrospective cohort study was conducted using the Longitudinal Health Insurance Database containing the records of 2 million individuals retrieved from Taiwan’s National Health Insurance Research Database. Fine–Gray competing risk analysis was used to determine the risk for the development of dementia in the toxoplasmosis cohort relative to the non-toxoplasmosis cohort. A sensitivity analysis was also conducted. The effects of antibiotics (sulfadiazine or clindamycin) on the risk of dementia were also analyzed.

**Results:**

We enrolled a total of 800 subjects, and identified 200 patients with toxoplasmosis and 600 sex- and age-matched controls without toxoplasmosis infection in a ratio of 1:3, selected between 2000 and 2015. The crude hazard ratio (HR) of the risk of developing dementia was 2.570 [95% confidence interval (CI) = 1.511–4.347, *P* < 0.001]. After adjusting for sex, age, monthly insurance premiums, urbanization level, geographical region, and comorbidities, the adjusted HR was 2.878 (95% CI = 1.709–4.968, *P* < 0.001). Sensitivity analysis revealed that toxoplasmosis was associated with the risk of dementia even after excluding diagnosis in the first year and the first 5 years. The usage of sulfadiazine or clindamycin in the treatment of toxoplasmosis was associated with a decreased risk of dementia.

**Conclusions:**

This finding supports the evidence that toxoplasmosis is associated with dementia and that antibiotic treatment against toxoplasmosis is associated with a reduced risk of dementia. Further studies are necessary to explore the underlying mechanisms of these associations.

**Graphical Abstract:**

**Figure:**
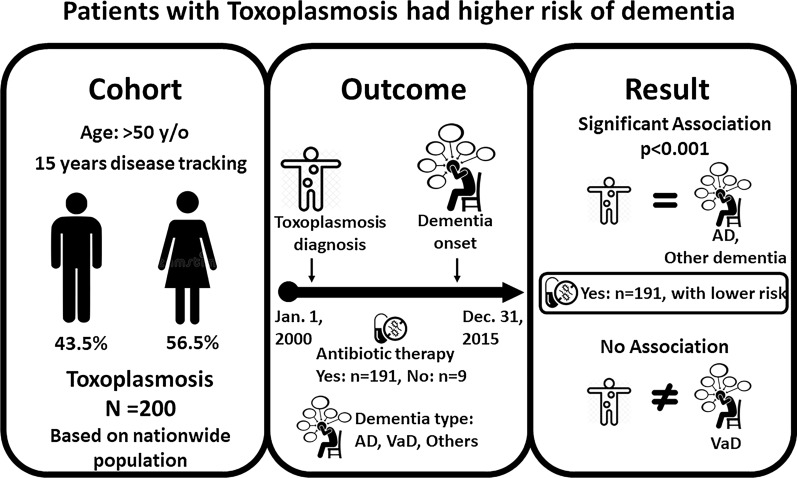

**Supplementary Information:**

The online version contains supplementary material available at 10.1186/s13071-021-04928-7.

## Background

Toxoplasmosis, which is caused by infection with the *Toxoplasma gondii* (*T. gondii*), parasite, affects about 25–30% of the population worldwide [1]. Individuals can become infected from the ingestion of tissue cysts, infected meat, or food contaminated with sporulated oocysts [1]. After ingestion, bradyzoites and sporozoites released from the cysts and oocysts eventually form tachyzoites [2], which can spread into the bloodstream and lymphatic system and cause distant organ invasion. The tachyzoites can then induce acute inflammation in the organs, which can lead to myocarditis, hepatitis, pneumonitis, or retinochoroiditis [2]. They can also cross the blood-brain barrier and invade the brain cells during the first week of infection [1]. In addition, chronic infection can occur by latent toxoplasmic cysts which remain in the tissues or the central nervous system (CNS) [1]. Elevated risk of cerebral toxoplasmosis has been noted in the elderly due to the possibility of increased immunosuppression [3]. In Taiwan, the Center for Disease Control lists the antibiotics sulfadiazine and clindamycin as treatment for toxoplasmosis [4].

Dementia is a common neurodegenerative disease characterized by symptoms of worsening cognition, emotional change, difficulties with language expression, and decreased motivation [5]. The most common cause is Alzheimer’s disease (AD), and other common causes include buildup of Lewy bodies, frontotemporal lobe degeneration, and vascular disease [6]. The pathophysiology of AD is of major concern and is related to the amyloid β (Aβ) protein and intracellular neurofibrillary tangles [7]. However, the inflammatory process can also be associated with neurodegenerative disorders such as AD, Parkinson’s disease (PD), Huntington’s disease (HD), amyotrophic lateral sclerosis (ALS), or multiple sclerosis (MS). Chronic inflammation can be induced by infectious agents including viruses (herpes simplex virus), bacteria (*Chlamydia pneumonia*), or parasites (*T. gondii*) [8, 9].

Several previous studies have shown that chronic toxoplasmic infection may be associated with human behavior alterations, obsessive-compulsive disorder, or even schizophrenia [10, 11]. Infection can also lead to other neurodegenerative symptoms including memory impairment [12] and cognitive decline [13]. The cysts’ location in the brain [14], the immune response [15], and changes in brain metabolism [16] can have effects on cognitive dysfunction. In recent years, two meta-analyses of several cross-sectional studies have reported results supporting an association between toxoplasmosis and the risk of AD [17, 18]. However, a case–control study by Mahami et al. (2016) found no significant relationship between toxoplasmosis and AD [19], and Perry et al. found no difference in serum *T. gondii* antibody titers between an AD group and control group with latent toxoplasmosis [20]. No previous nationwide cohort studies have investigated the association among toxoplasmosis, antibiotic treatment, and the risk of dementia. Therefore, we conducted the present study using Taiwan’s National Health Insurance Research Database (NHIRD) to investigate whether toxoplasmosis is associated with the risk of dementia, along with the role of antibiotic treatment in the risk of dementia in patients with toxoplasmic infections.

## Methods

### Design and study methods

This retrospective cohort study is based on the NHIRD, provided by the Health and Welfare Data Science Center (HWDC), Ministry of Health and Welfare (MOHW), Taiwan. The National Health Insurance (NHI) scheme was established in 1995, and as of June 2009, it included contracts with 97% of medical providers, with approximately 23 million people enrolled in the program, or more than 99% of the entire population [21, 22]. The details of this program have been documented in previous studies [23–30]. The NHIRD registration files and the original claims data include overall data on personal information and disease coding.

The NHIRD also records inpatient care, ambulatory care, dental care, and prescription drugs received by the insured and their date of birth. Pursuant to the Personal Information Protection Act, individual identifiers are encrypted before the release of data for research. In the present study, patients diagnosed with toxoplasmosis during the period 2000–2015 were enrolled, and those recorded in the NHI program were coded according to the International Classification of Diseases, Ninth Revision, Clinical Modification (ICD-9-CM), as ICD-9 codes of 130.

All diagnoses of dementia were made by board-certified psychiatrists or neurologists. Toxoplasmosis was confirmed by serum antibody screening and avidity test or polymerase chain reaction (PCR) [4]. Several previous studies have revealed high accuracy and validity of the diagnoses in the NHIRD [31–33], and licensed medical records technicians verify the coding before reimbursement claims can proceed for hospitals and clinics [34]. Furthermore, the NHI Administration appoints several senior external specialists in psychiatry, neurology, infectious disease, and other related medical specialties for random review of the records of ambulatory care visits and inpatient claims to verify the accuracy of the diagnoses [35]. Thus, the NHIRD records are suitable for examining the longitudinal association between toxoplasmosis and the potential risk of subsequent development of dementia.

### Ethical approval

This study was approved by the Institutional Review Board of the Tri-Service General Hospital (TSGH IRB No. 2-107-05-026). Because the patient identifiers were encrypted before their data were used for research purposes in order to protect confidentiality, the requirement for written or verbal consent from patients for data linkage was waived.

### Study population

**Fig. 1:**
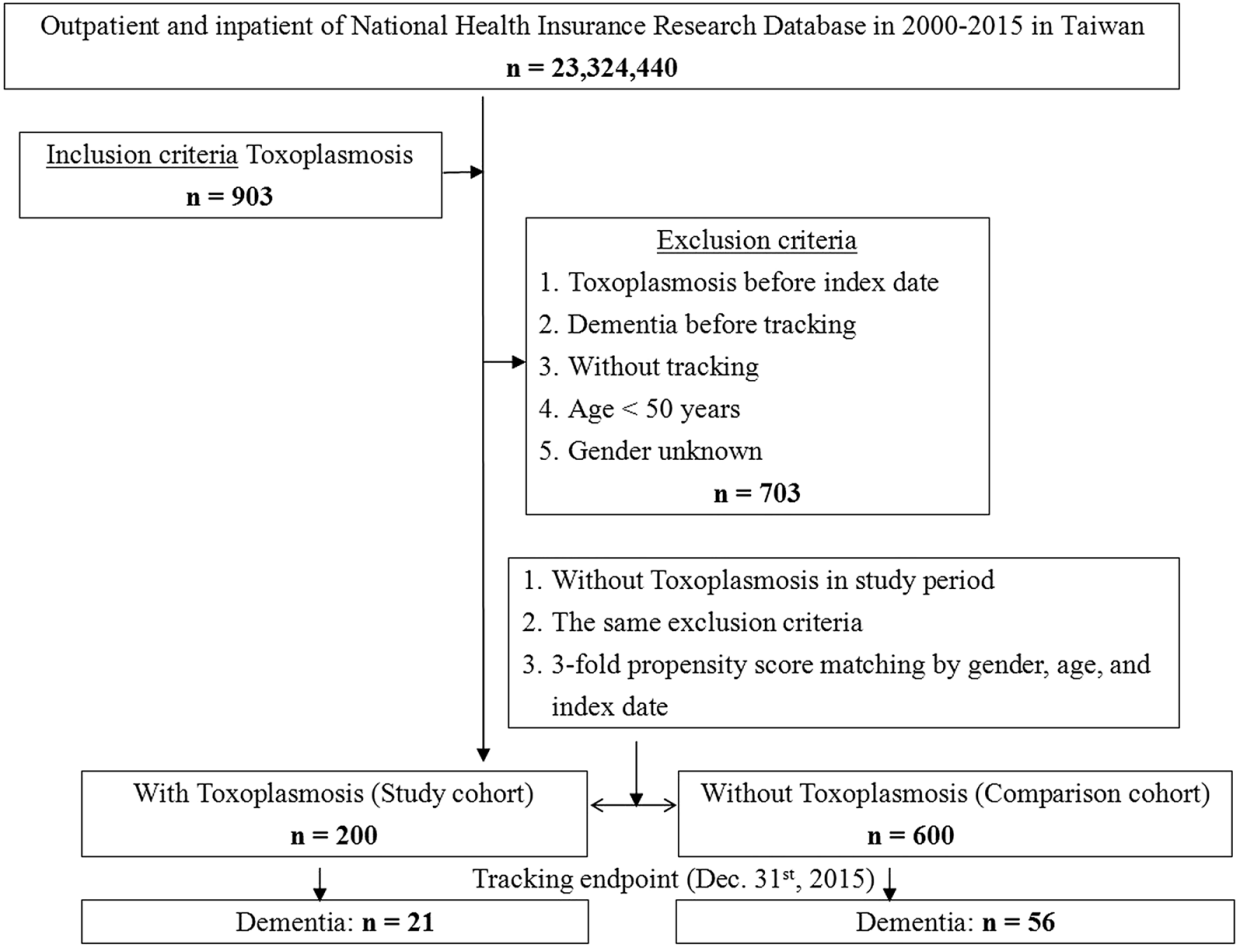
The flowchart of study sample selection

This was a retrospective cohort study. From the Longitudinal Health Insurance Database (LHID) including 2 million individuals, a randomly stratified sub-database retrieved from the NHIRD was used to identify individuals ≥ 50 years of age with a diagnosis of toxoplasmosis during the period between January 1, 2000, and December 31, 2015, according to ICD-9-CM code 130.x. In this 15-year follow-up study, patients were excluded if they were diagnosed with dementia or toxoplasmosis before 2000, were diagnosed with dementia before the first visit for toxoplasmosis, or were aged < 50. The date of toxoplasmosis diagnosis was defined as the index date. Figure 1 depicts the flowchart of this study for the comparison of patients with toxoplasmosis and controls. In addition, a flowchart of the study for the comparison of patients with toxoplasmosis with and without antibiotic treatment is presented in Additional file 1: Figure S1.

### Covariates

The covariates included sex, age group (50–64, ≥ 65 years), geographical area of residence (northern, central, southern, or eastern Taiwan), urbanization level of residence (levels 1 to 4), levels of hospitals as medical centers, regional or local hospitals, and monthly income (in New Taiwan dollars [NT$]: < 18,000, 18,000–34,999, ≥ 35,000). The urbanization level of residence was defined according to the population, along with various indicators of the level of political, economic, cultural, and metropolitan development. Level 1 was defined as a population of > 1,250,000, and a specific designation of significant political, economic, cultural, and metropolitan development. Level 2 was defined as a population between 500,000 and 1,249,999, and as playing an important role in the political system, economy, and culture. Urbanization levels 3 and 4 were defined as a population between 149,999 and 499,999, and < 149,999, respectively.

The comorbidities in this study were diabetes mellitus (DM), hypertension, hyperlipidemia, coronary artery disease (CAD), human immunodeficiency virus (HIV) infection/acquired immunodeficiency syndrome (AIDS), and other immune deficiency diseases. All the ICD codes of the comorbidities are as listed in Additional file 3: Table S1.

Data on the usage of the antibiotics sulfadiazine and clindamycin were collected. The data on the defined daily dose (DDD) were obtained from the WHO Collaborating Centre for Drug Statistics Methodology (https://www.whocc.no/), and the duration of antibiotics usage was calculated by dividing the cumulative doses by the DDD of the antibiotics. We analyzed the effects on the risk of dementia between the two subgroups with or without the antibiotics treatment, with the sample divided by the covariates with the references of previous studies using the NHIRD, regarding the treatment effects of medications [36–38]. The yearly times of the visits to psychiatry, neurology, and infection medicine clinics were also recorded.

### Study outcomes

All the participants were followed from the index date until the onset of dementia, withdrawal from the NHIRD, or the end of 2015. The patients with dementia were grouped into those with Alzheimer’s disease (AD), vascular dementia (VaD), or other types of dementia. At least three visits in one consecutive year in the NHIRD records would be regarded as a diagnosis of dementia. All the ICD codes of dementia are as listed in Additional file 3: Table S1.

### Statistical analysis

All analyses were performed using SPSS software version 22 (IBM Corp., Armonk, NY, USA). Chi-square and *t*-tests were used to evaluate the distribution of the categorical and continuous variables, respectively. The Fisher exact test for categorical variables was used to statistically examine the differences between the two cohorts. Fine–Gray survival analysis was used to determine the risk of dementia, and the results are presented as a hazard ratio (HR) with a 95% confidence interval (CI). A sensitivity analysis excluding the diagnosis of dementia both within the first year and the first 5 years was conducted to avoid protopathic bias. The difference in the risk of dementia between the toxoplasmosis subjects and control groups was estimated using the Kaplan–Meier method with the log-rank test. A two-tailed *P*-value < 0.05 was considered to indicate statistical significance.

## Results

### Sample characteristics

Table 1 shows that a total of 800 patients were enrolled, including 200 subjects with toxoplasmosis and 600 controls without toxoplasmosis, which were matched 1:3 for age, sex, and index year. There were no differences in sex or age. The toxoplasmosis cohort tended to have a higher percentage of comorbidities of DM, but a slightly lower percentage of HIV/AIDS and other immunodeficiency diseases, in comparison to the non-toxoplasmosis controls. The patients with toxoplasmosis tended to have monthly insurance premiums of NT$18,000–34,999, lived in central and eastern Taiwan and the outlying islands, resided in areas of level 2, 3, and 4 urbanization, and sought medical care from the medical center and regional hospital. In addition, patients with toxoplasmosis visited more clinics of infectious disease and neurology than the control group.

**Table 1:** Characteristics of study population at the baseline

Toxoplasmosis	With		Without		*P*-value*
Variables	*n*	%	*n*	%	
Total	200	25.00	600	75.00	
Gender					0.999
Male	87	43.50	261	43.50	
Female	113	56.50	339	56.50	
Age (years)	62.11 ± 8.88		63.18 ± 8.71		0.135
Age groups (years)					0.999
50–64	131	65.50	393	65.50	
≧65	69	34.50	207	34.50	
Insurance premium (NT$)					< 0.001
< 18,000	143	71.50	593	98.83	
18,000–34,999	48	24.00	7	1.17	
≧35,000	9	4.50	0	0	
Diabetes mellitus					0.014
Without	179	89.50	493	82.17	
With	21	10.50	107	17.83	
Hypertension					0.482
Without	155	77.50	479	79.83	
With	45	22.50	121	20.17	
Hyperlipidemia					0.357
Without	196	98.00	578	96.33	
With	4	2.00	22	3.67	
Coronary artery disease					0.885
Without	183	91.50	547	91.17	
With	17	8.50	53	8.83	
Obesity					-
Without	200	100	600	100	
With	0	0	0	0	
HIV					< 0.001
Without	196	98.00	596	99.33	
With	4	2.00	4	0.67	
Immune deficiency					< 0.001
Without	190	95.00	589	98.17	
With	10	5.00	11	1.83	
CCI_R	0.76 ± 1.84		0.69 ± 1.48		0.596
Location					< 0.001
Northern Taiwan	68	34.00	244	40.67	
Central Taiwan	96	48.00	162	27.00	
Southern Taiwan	26	13.00	170	28.33	
Eastern Taiwan	8	4.00	22	3.67	
Outlying islands	2	1.00	2	0.33	
Urbanization level					0.003
1 (highest)	53	26.50	208	34.67	
2	96	48.00	260	43.33	
3	15	7.50	39	6.50	
4 (lowest)	36	18.00	93	15.50	
Level of care					0.002
Hospital center	81	40.50	220	36.67	
Regional hospital	75	37.50	172	28.67	
Local hospital	44	22.00	208	34.67	
Department of visits in study period					
Infection	1.12 ± 1.30		0.85 ± 1.08		0.004
Neurology	2.54 ± 2.69		2.03 ± 2.51		0.015
Psychiatry	3.01 ± 3.47		2.75 ± 2.83		0.289

*Chi-square/Fisher exact test for categorical variables and *t*-test for continuous variables, *CCI_R* Charlson Comorbidity Index, dementia and HIV removed

### Kaplan–Meier model for the cumulative incidence of dementia

**Fig. 2:**
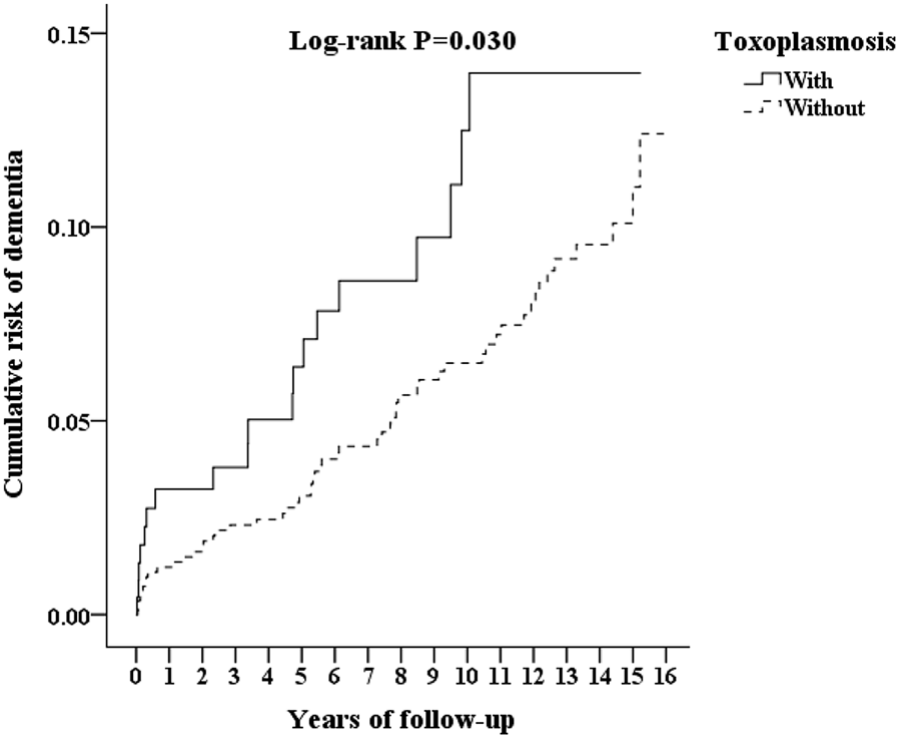
Kaplan–Meier plot for cumulative incidence of dementia stratified by toxoplasmosis with log-rank test

Of the toxoplasmosis patients, 21/200 (457.84 per 10^5^ person-years) developed dementia, as compared to 56/600 (323.42 per 10^5^ person-years) in the control group, and the difference was statistically significant in Kaplan–Meier survival analysis (log-rank, *P* = 0.030, Fig. 2).

Of the toxoplasmosis patients, 20/191 with antibiotic treatment (455.72 per 10^5^ person-years) developed dementia, as compared to 1/9 without antibiotic treatment (507.67 per 10^5^ person-years) in the control group, and the difference was statistically significant in the Kaplan–Meier survival analysis (log-rank, *P* = 0.099, Additional file 2: Figure S2).

### HR analysis of dementia in patients with toxoplasmosis

Table 2 shows that Fine–Gray competing risk model analysis revealed that the study subjects were more likely to develop psychiatric disorders (crude hazard ratio [HR]: 2.570, 95% CI = 1.511–4.347, *P* < 0.001). After adjusting for gender, age, monthly insurance premiums, urbanization level, geographical region, and comorbidities, the adjusted HR was 2.878 (95% CI = 1.709–4.968, *P* < 0.001). Male gender and higher Charlson Comorbidity Index (CCI) were associated with a higher risk of developing dementia. The toxoplasmosis patients aged ≥ 65 years were associated with a higher risk of developing dementia, in comparison to the patients aged 50–64.

**Table 2:** Factors of dementia using Cox regression and the Fine–Gray competing risk model

	No competing risk in the model				Competing risk in the model			
Variables	Adjusted HR	95% CI	95% CI	*P*-value*	Adjusted HR	95% CI	95% CI	*P*-value*
Toxoplasmosis (reference: without)	2.570	1.511	4.347	< 0.001	2.878	1.709	4.968	< 0.001
Male (reference: female)	1.841	1.123	3.018	0.001	1.989	1.235	3.310	< 0.001
Age ≥ 65 (reference: age 40–59)	1.685	1.240	2.006	< 0.001	1.703	1.259	2.034	< 0.001
CCI_R	1.286	1.032	1.537	0.029	1.335	1.050	1.682	0.007

*Chi-square/Fisher exact test for categorical variables and *t*-test for continuous variables, *HR* hazard ratio, *CI* confidence interval, *Adjusted HR* adjusted variables listed in the Table 1, *CCI_R* Charlson Comorbidity Index, dementia and HIV removed

### Types and sensitivity analysis of dementia after toxoplasmosis

Table 3 reveals that toxoplasmosis was associated with overall dementia, AD, and other degenerative dementia, with adjusted HR of 2.878 (*P* < 0.001), 6.675 (*P* < 0.001), and 3.162 (*P* < 0.001), respectively. Toxoplasmosis was noted as being associated with VaD. Table 3 also shows that, after the exclusion of diagnosis within the first year or first 5 years, toxoplasmosis was only associated with other degenerative dementia.

**Table 3:** Factors of dementia subgroup and sensitivity test using Cox regression

	Toxoplasmosis	With			Without			Competing risk in the model			
Sensitivity test	Dementia subgroup	Events	Rate (per 10^5^ PYs)		Events	Rate (per 10^5^ PYs)		Adjusted HR	95% CI	95% CI	*P*-value*
Overall	Overall	21	457.84		56	323.42		2.878	1.709	4.968	< 0.001
	AD	1	21.80		1	5.78		6.675	3.157	11.241	< 0.001
	VaD	1	21.80		9	51.98		0.956	0.312	1.478	0.570
	Other degenerative dementia	19	414.24		46	265.67		3.162	1.875	5.406	< 0.001
In the first year excluded	Overall	15	352.13		47	277.21		2.538	1.345	4.497	< 0.001
	AD	0	0		0	0		-	-	-	-
	VaD	0	0		7	41.29		0.000	-	-	0.976
	Other dementia	15	352.13		40	235.92		2.973	1.801	5.213	< 0.001
In the first 5 years excluded	Overall	8	219.94		33	239.32		1.862	1.045	3.270	0.007
	AD	0	0		0	0		-	-	-	-
	VaD	0	0		4	29.01		0.000	-	-	0.983
	Other degenerative dementia	8	219.94		29	210.31		2.011	1.099	3.452	0.001

*Chi-square/Fisher exact test for categorical variables and *t*-test for continuous variables, *PYs* person-years, *Adjusted HR* adjusted hazard ratio (adjusted for the variables listed in Table 3), *CI* confidence interval

### The effects of antiprotozoal medications for toxoplasmosis and the risk of toxoplasmosis

 Antiprotozoal medication usage for toxoplasmosis was associated with a lower risk than that in the comparison group. Both sulfadiazine and clindamycin, either monotherapy or combination treatment, were associated with a lower risk of dementia (Table 4).

**Table 4:** Factors of dementia among different models using Cox regression and the Fine–Gray competing risk model

Antibiotics	Toxoplasmosis subgroup	Events	PYs	Rate (per 10^5^ PYs)	Competing risk in the model			
					Adjusted HR	95% CI	95% CI	*P*-value*
Sulfadiazine	With toxoplasmosis, without sulfadiazine	5	1015.10	492.56	Reference			
	With toxoplasmosis, with sulfadiazine	16	3571.66	447.97	0.909	0.887	0.932	0.013
	With toxoplasmosis, with sulfadiazine < 30 days	5	1047.15	477.48	0.967	0.943	0.991	0.046
	With toxoplasmosis, with sulfadiazine 30–364 days	5	1257.18	397.72	0.808	0.782	0.845	< 0.001
	With toxoplasmosis, with sulfadiazine ≥ 365 days	6	1267.33	473.44	0.932	0.901	0.986	0.041
Clindamycin	With toxoplasmosis, without clindamycin	5	879.28	568.65	Reference			
	With toxoplasmosis, with clindamycin	16	3707.48	431.56	0.759	0.732	0.797	< 0.001
	With toxoplasmosis, with clindamycin < 30 days	5	1048.48	476.88	0.832	0.784	0.863	< 0.001
	With toxoplasmosis, with clindamycin 30–364 days	5	1056.84	473.11	0.821	0.776	0.859	< 0.001
	With toxoplasmosis, with clindamycin ≥ 365 days	6	1602.17	374.49	0.664	0.602	0.692	< 0.001
Sulfadiazine	With toxoplasmosis, without antibiotics	1	198.15	504.67	Reference			
and / or	With toxoplasmosis, with any antibiotics	20	4388.61	455.72	0.927	0.894	0.950	0.022
Clindamycin	With toxoplasmosis, with any antibiotics < 30 days	5	1030.45	485.22	0.970	0.942	0.995	0.047
	With toxoplasmosis, with any antibiotics 30–364 days	7	1544.20	453.31	0.965	0.931	0.983	0.041
	With toxoplasmosis, with any antibiotics ≥ 365 days	8	1813.96	441.02	0.864	0.803	0.899	< 0.001
	With toxoplasmosis, with both antibiotics	12	2890.53	415.15	0.812	0.694	0.872	< 0.001
	With toxoplasmosis, with both antibiotics < 30 days	5	1065.18	469.40	0.853	0.793	0.901	< 0.001
	With toxoplasmosis, with both antibiotics 30–364 days	3	769.82	389.70	0.750	0.615	0.830	< 0.001
	With toxoplasmosis, with both antibiotics ≥ 365 days	4	1055.54	378.95	0.721	0.594	0.825	< 0.001

*Chi-square/Fisher exact test for categorical variables and *t*-test for continuous variables, *PYs* person-years, *Adjusted HR* adjusted hazard ratio (adjusted for the variables listed in Table 1), *CI* confidence interval

## Discussion

In this retrospective cohort study, there are several noteworthy findings. First, patients with toxoplasmosis had a nearly 2.8-fold increased risk of developing dementia. After the sensitivity analysis, excluding the diagnosis of dementia for the first year and the first 5 years after toxoplasmosis was diagnosed, patients with toxoplasmosis still had a twofold increased risk for developing dementia. Second, the sensitivity analysis revealed that, after excluding the AD diagnosis in the first year and first 5 years after toxoplasmosis, the association became insignificant, but other types of degenerative dementia were still associated with toxoplasmosis. However, other types of degenerative dementia were found to be proportionately higher than AD and VaD, and most of the community studies revealed that Alzheimer-type dementia is the most common cause of dementia in Taiwan (40–60% of all dementias), followed by vascular dementia (20–30% of all dementias) and mixed or other dementias (7–15%) [39–41]. One possible explanation for this disparity is that some subjects were classified as having other degenerative types of dementia, similar to the findings of previous studies [34, 37]. Third, the usage of the medications sulfadiazine and clindamycin, either in monotherapy or combination treatment, were associated with a lower risk of dementia. To the best of our knowledge, this is the first nationwide, population-based study to investigate the association between toxoplasmosis and the risk of dementia and the effects of antibiotic usage in reducing risk after toxoplasmosis infections.

We also discovered that there were no significant differences in the ratios between the two cohorts (21/200 vs. 56/600), or differences between the treatment and non-treatment groups (20/191 vs. 1/9). The difference was only apparent when the duration of follow-up after the exposure to toxoplasmosis was considered, that is, the person-years. This implies that toxoplasmosis might not be the direct cause of dementia, but it could accelerate the process, resulting in early onset of dementia. In addition, antibiotic treatment could attenuate this process. This might indicate that chronic inflammation, instead of the toxoplasmic infection itself, contributes to the process in the development of dementia.

In previous studies, the brain was found to be the main target organ in *T. gondii* infection, and may cause life-threatening encephalitis in immunocompromised patients [42]. In healthy individuals, the tachyzoites of the parasite can be cleaned by the cellular immune response in the proliferative stage of the systemic infection [43]. In an infected mouse brain model, interferon-gamma (IFN-γ) produced by lymphocytes, microglial cells, and blood-derived macrophages mediated the cell immune response to the proliferating tachyzoites [44]. In addition, IFN-γ was found to activate astrocytes which inhibit tachyzoite replication by nitric oxide (NO) production [44]. Microglia are also resident innate immune cells in the CNS and are the main cause of the inflammatory process. Uncontrolled activation of microglial cells may cause neurotoxicity due to the release of inflammatory cytokines, NO, or superoxide (SOD). In addition to acute toxoplasmosis caused by tachyzoites, bradyzoites of the parasite can produce a tissue cyst and slowly replicate in the brain or muscles, leading to latent toxoplasmosis [43]. Although tachyzoites can induce more obvious inflammatory cytokine production than bradyzoites [44], the dormant parasite can resume pathogenic activity and kill a host with immune deficiency. Latent toxoplasmosis is asymptomatic in normal conditions. However, in contrast to acute toxoplasmosis, latent toxoplasmosis might cause a slow and cumulative effect that decreases psychomotor performance [45]. Early animal models already demonstrated pathological changes in the cyst-containing region of the brain in mice, including the granulomatous change in the perivascular areas and necrotic tissue deposition with vascular sclerosis [46].

Torres et al. designed another mouse model and argued that the study, by Möhle et al., has not evaluated the advanced signs as *T. gondii*-driven cerebral amyloid angiopathy (CAA). In addition, there was also Aβ immunoreactivity co-localized with the *T. gondii* cysts as early as day 15 post-infection and widespread Aβ immunoreactivity. They were detected in other areas of the brain where they did not co-localize with cysts at days 60 to 90 post-infection [47]. Moreover, Torres et al. pointed out that Aβ immunoreactivity may lead to *N*-methyl-*d*-aspartate receptor (NMDAR) loss. In the CNS, glutamate plays a role in neuron excitation and could be endocytosed or released at the synapse through NMDAR on neural cells. Therefore, NMDAR plays an important role in the synaptic connection which controls the function of learning and memory. NMDAR dysfunction is strongly associated with AD [48]. There was also strong evidence that an NMDAR antagonist could prevent neuronal dysfunction through Aβ immunoreactivity [49]. However, some studies found that countries with high seropositivity of *T. gondii* did not have a higher prevalence of AD. For example, in the 1970s, the seroprevalence in France was 70% [1], but only 3% prevalence of AD was noted in people older than 60 years in 2012 [50]. Möhle et al. (2016) reported a mouse model study and discovered that there were reduced Aβ plaques in *T. gondii*-infected mice compared to the non-infected mice [51]. The association between toxoplasmosis and AD, as well as the underlying mechanisms, has yet to be clarified.

Our study has several strengths: First, we used Taiwan’s NHIRD, which is a valuable resource to address this issue in a nationwide population. Second, several previous studies have demonstrated the accuracy and validity of several diagnoses of neuropsychiatric disorders in the NHIRD, such as Tourette syndrome [52], stroke [31, 53–55], sleep apnea [56], and major depressive disorder [57]. In addition, as previously mentioned, the in-hospital licensed medical records technicians and the NHI Administration would have verified the diagnoses in the claims dataset [22, 35] for the diagnosis. Third, previous studies have also demonstrated concordance between Taiwan’s National Health Survey and the NHIRD on a variety of diagnoses [58], medication usage [58], and health system utilization [58, 59]. Therefore, this study was conducted using a large, nationwide, and reliable database for the association between toxoplasmosis and psychiatric morbidities in an Asian country.

The present study has several limitations that warrant consideration. First, similar to previous studies, not all data were recorded in the NHIRD, and we were unable to evaluate family history, neurological severity, types, laboratory parameters, the availability of rehabilitation, or additional examination findings (e.g., neuroimaging). Therefore, the lack of data on the clinical and radiological course and treatment of the disease was a limitation. Second, other factors, such as genetic, psychosocial, and environmental factors, were not included in the dataset. However, the present study covers all of Taiwan’s hospitals and > 99% of the Taiwanese population during a 15-year period, thereby increasing the likelihood that our data are valid and representative. Third, the recorded prevalence of *Toxoplasma* infection in Taiwan was about 10% in 2006 (https://nidss.cdc.gov.tw), but this was with a focus only on pregnant women, and it did not represent the general prevalence in the total population. Therefore, we were not able correlate the current prevalence of dementia with the prevalence of toxoplasmosis in the past. Fourth, there were very few non-treatment patients in our study, and we used a one-sided test to analyze the significance between the treatment and non-treatment groups. This limits the generalizability of the results regarding antibiotic effects and their association with reduced risk of dementia. Further evaluations are needed using randomized clinical trials or observational studies in a larger population.

## Conclusions

To the best of our knowledge, we have provided the first evidence that toxoplasmosis is associated with dementia in Taiwan. The results show that the usage of antibiotics for toxoplasmosis might be beneficial in attenuating the risk of dementia for patients with toxoplasmosis. Clinicians should focus more attention on the risk of dementia in patients with toxoplasmosis.

## Supplementary Information

**Additional file 1**:** Figure S1**. The flowchart for the comparison of patients with toxoplasmosis with and without antibiotic treatment

**Additional file 2**:** Figure S2**. The Kaplan-Meier survival analysis in toxoplasmosis patients with or without antibiotic treatment. In the comparison of the two groups, the difference was statistically significant

**Additional file 3**:** Table S1**. International Classification of Diseases, Ninth Revision, Clinical Modification codes in this study

## Data Availability

The datasets on the study population that were obtained from the NHIRD (http://nhird.nhri.org.tw/en/index.html) are maintained in the NHIRD (http://nhird.nhri.org.tw/). The National Health Research Institutes (NHRI) is a nonprofit foundation established by the government. Only citizens of Taiwan who fulfill the requirements for conducting research projects are eligible to apply for the NHIRD. The use of the NHIRD is limited to research purposes only. Applicants must follow the Computer-Processed Personal Data Protection Act (http://www.winklerpartners.com/?p=987) and the related regulations of the National Health Insurance Administration and NHRI, and an agreement must be signed by the applicant and their supervisor upon application submission. All applications are reviewed for approval of data release.

## Abbreviations

Aβ: Amyloid β
AD: Alzheimer’s disease
AIDS: Acquired immune deficiency syndrome
ALS: Amyotrophic lateral sclerosis
CAA: Cerebral amyloid angiopathy
CAD: Coronary artery disease
CI: Confidence interval
CNS: Central nervous system
DDD: Defined daily dose
DM: Diabetes mellitus
HD: Huntington’s disease
HIV: Human immunodeficiency virus
HR: Hazard ratio
HWDC: Health and Welfare Data Science Center
ICD-9-CM: International Classification of Disease, Ninth Revision, Clinical Modification
IFN-γ: Interferon-gamma
LHID: Longitudinal Health Insurance Database
MOHW: Ministry of Health and Welfare
MS: Multiple sclerosis
NHI: National Health Insurance
NHIRD: National Health Insurance Research Database
NMDAR: *N*-methyl-d-aspartate receptor
NO: Nitric oxide
NT$: New Taiwan dollars
PCR: Polymerase chain reaction
PD: Parkinson’s disease
SOD: Superoxide
*T. gondii*: *Toxoplasma gondii*
VaD: Vascular dementia
