# Supplementary figures and images for: Risk of dementia in patients with toxoplasmosis: a nationwide, population-based cohort study in Taiwan
Source: Parasit Vectors. 2021 Aug 28;14:435. doi: 10.1186/s13071-021-04928-7 (PMC8401101; doi:10.1186/s13071-021-04928-7)

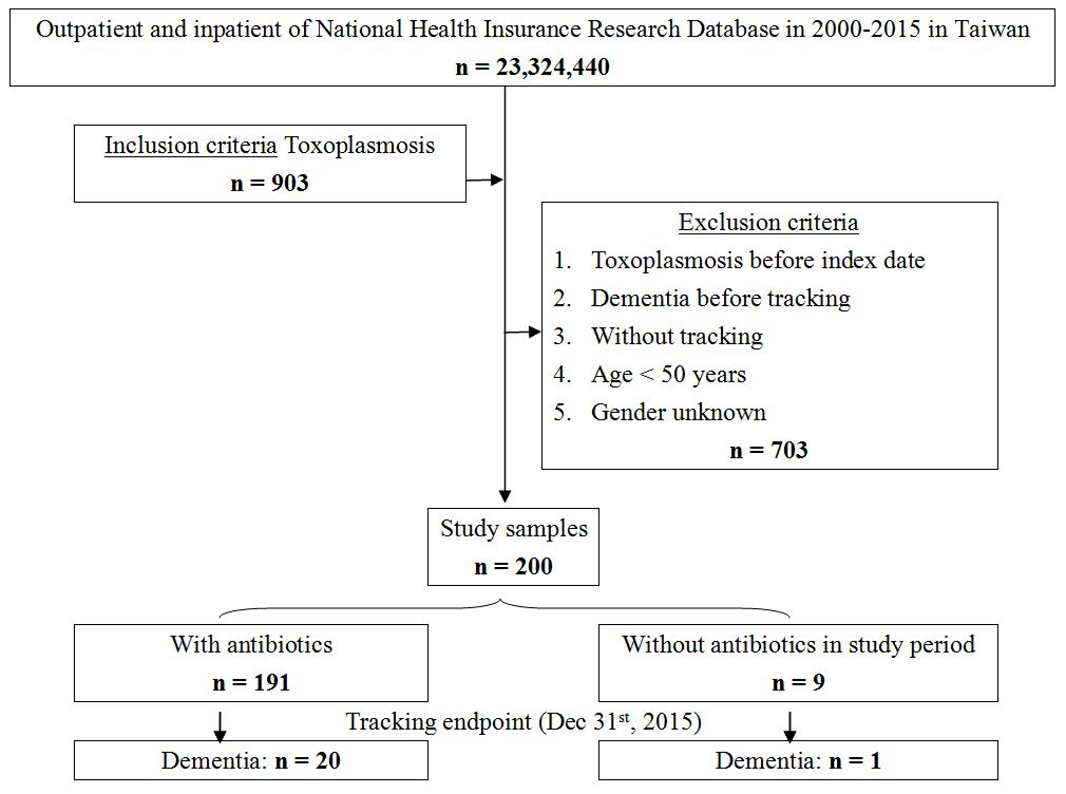

Supplement: Supplementary file 1 — Additional file 1: Figure S1. The flowchart for the comparison of patients with toxoplasmosis with and without antibiotic treatment [file 13071_2021_4928_MOESM1_ESM.tiff]

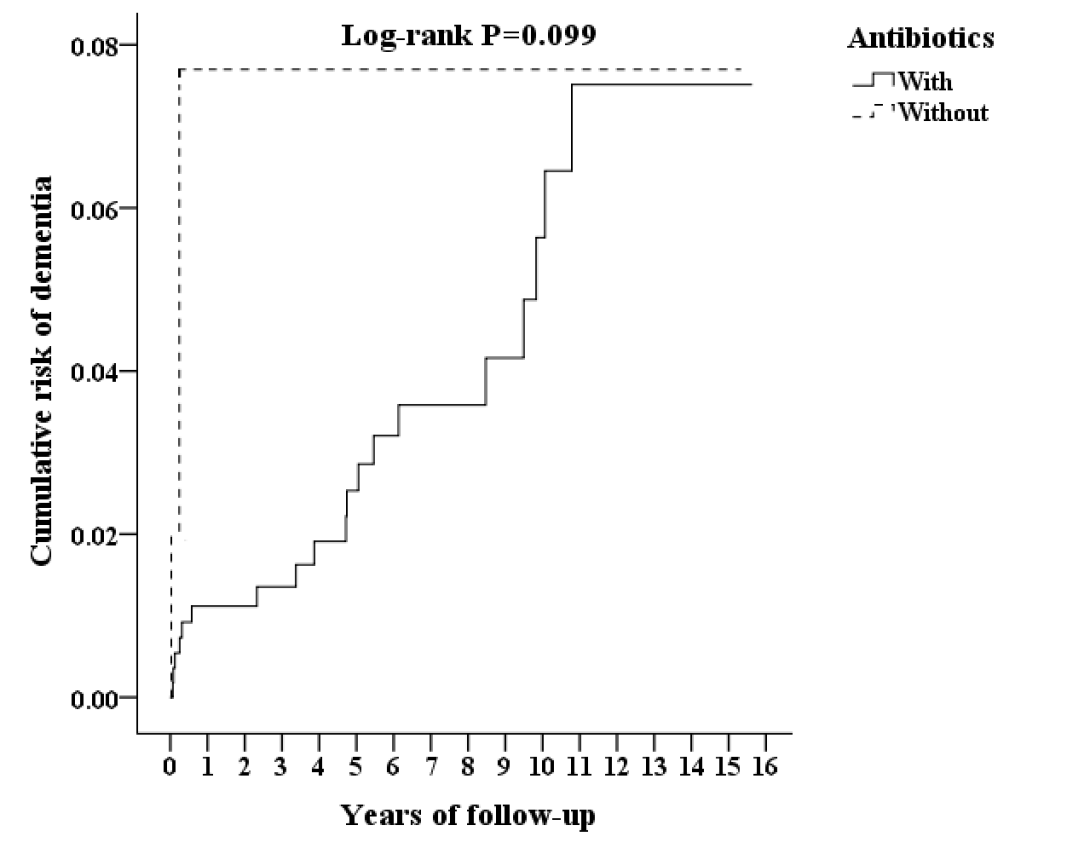

Supplement: Supplementary file 2 — Additional file 2: Figure S2. The Kaplan-Meier survival analysis in toxoplasmosis patients with or without antibiotic treatment. In the comparison of the two groups, the difference was statistically significant [file 13071_2021_4928_MOESM2_ESM.tiff]
