# Supplementary material for: Risk of dementia in patients with toxoplasmosis: a nationwide, population-based cohort study in Taiwan
Source: Parasit Vectors. 2021 Aug 28;14:435. doi: 10.1186/s13071-021-04928-7 (PMC8401101; doi:10.1186/s13071-021-04928-7)
Supplement: Supplementary file 3 — Additional file 3: Table S1. International Classification of Diseases, Ninth Revision, Clinical Modification codes in this study [file 13071_2021_4928_MOESM3_ESM.docx]

| **Table S1. International Classification of Diseases, Ninth Revision, Clinical Modification codes in this study** | |
| --- | --- |
|  | **ICD-9-CM codes** |
| **Study population:** Toxoplasmosis | 130 |
| Dementia | 290.0, 290.10-290.13, 290.20-290.21, 290.3. 290.40-290.43, 290.8-290.9, 331.0 |
| Alzheimer's disease | 331.0 |
| Vascular dementia | 290.4 |
| Other degenerative dementia | 290.x except 290.4 |
| **Comorbidities** |  |
| Diabetes mellitus | 250 |
| Hypertension | 401.1, 401.9, 402.10, 402.90, 404.10, 404.90, 405.1, 405.9 |
| Hyperlipidemia | 272 |
| Coronary artery disease | 410-414 |
| Human immunodeficiency virus infections/ acquired immune deficiency syndrome | 042, 043, 044, plus viral loads or CD4+ tests as procedure codes, 12071A, 12071B, 12073A, 12073B, 12074A, 12074B, 14074B, |
| Other immune deficiency diseases | 279 |
| **ICD-9-CM: International Classification of Diseases, Ninth Revision, Clinical Modification;**  **CD 4+: Cluster of Differentiation 4 positive** | |
